# The platelet-to-high-density lipoprotein cholesterol ratio as a potential biomarker linking tobacco exposure to accelerated reproductive aging in women: A cross-sectional study

**DOI:** 10.1097/MD.0000000000050198

**Published:** 2026-08-28

**Authors:** Jie Liao, De Qiong, Dawa Zhuoma, Weiwei Ning, Da Ga, Silang Quncuo, Jing Jing, Lingxiu Li, Yalan Li, Xin Li, Bin Lv, Hengxi Chen

**Affiliations:** a Department of Obstetrics and Gynecology, Tibet Autonomous Region Women’s and Children’s Hospital, West China Second University Hospital of Sichuan University, Lhasa, China; b Department of Obstetrics and Gynecology, West China Second University Hospital, Sichuan University, Chengdu, Sichuan, China.

**Keywords:** NHANES, platelet-to-high-density lipoprotein cholesterol ratio, reproductive aging, serum cotinine, tobacco exposure

## Abstract

Female reproductive aging, commonly reflected by menopausal age and reproductive lifespan, is influenced by environmental exposures and metabolic health, and tobacco exposure has been associated with earlier menopause and shortened reproductive lifespan. Because this association may involve inflammatory activation and lipid-metabolic alterations, the platelet-to-high-density lipoprotein cholesterol ratio (PHR) may provide a useful composite marker for evaluating tobacco-related reproductive aging. We analyzed data from 6048 women in the US National Health and Nutrition Examination Survey (1999–2018). Serum cotinine and PHR were ln-transformed. Weighted multivariable linear regression assessed associations with menopausal age and reproductive lifespan, adjusting for demographic, lifestyle, and clinical covariates. Exploratory statistical mediation analysis was used to examine the extent to which PHR was statistically related to the association between cotinine and reproductive aging. Restricted cubic splines explored dose-response patterns and possible threshold effects. Higher serum cotinine and PHR were independently associated with earlier menopause (β = −0.28 and −1.86) and shorter reproductive lifespan (β = −0.26 and −1.91; all *P* < .001). A modest statistically mediated proportion attributed to PHR was observed, accounting for 5.69% to 6.08% of the cotinine-related association. Dose-response analyses suggested possible thresholds (ln-cotinine > −3.47; ln-PHR > 5.13), above which adverse associations became more apparent. Associations were stronger among younger, non-Hispanic White, nondiabetic, and non-hypertensive women. Our study suggests that PHR may be a potentially useful biomarker associated with female reproductive aging. Higher serum cotinine and PHR were associated with earlier menopause and shorter reproductive lifespan, and PHR showed a modest, statistically mediated proportion in these associations. The observed thresholds may be useful primarily for hypothesis generation and future validation, but prospective or longitudinal studies are needed to clarify the potential role of PHR in reproductive aging.

## 1. Introduction

Female reproductive aging is clinically characterized by menopausal age and reproductive lifespan. It constitutes more than mere cessation of fertility and represents a critical transition point for subsequent health trajectories. Substantial evidence links this biological process to elevated risks of cardiovascular pathologies, osteoporosis, and neurocognitive decline, positioning it as a pivotal determinant of women’s healthspan.^[1–3]^ This multifaceted phenomenon arises from the interplay of intrinsic factors, including genetic predispositions, oxidative stress, mitochondrial dysfunction, and chromosomal instability, and modifiable extrinsic influences, most notably environmental exposures and metabolic perturbations.^[4,5]^ Critically, tobacco smoke, pollutants, atherogenic dyslipidemia, and chronic inflammation accelerate ovarian aging through synergistic pathways, underscoring the emerging paradigm of metabolic-environmental crosstalk in reproductive senescence.^[6,7]^

Tobacco exposure, whether active or passive, has been consistently associated with diminished ovarian reserve and earlier menopause, though its mechanistic underpinnings remain incompletely elucidated. A growing body of epidemiological evidence has shown that smoking is associated with earlier natural menopause, with stronger associations observed among women with greater smoking intensity or longer duration of exposure. In population-based studies, serum cotinine is widely used as an objective biomarker of recent tobacco exposure because of its sensitivity and practicality, although its relatively short biological half-life requires cautious interpretation when it is used to reflect longer-term smoking patterns.^[8–10]^ Concurrently, the platelet-to-high-density lipoprotein cholesterol (HDL-C) ratio (PHR) has gained attention as an integrative metric reflecting metabolic-inflammatory dysregulation in aging and women’s reproductive health. By combining platelet (PLT) activation with its pro-thrombotic and pro-inflammatory properties, and HDL functionality, including anti-inflammatory and cholesterol transport activities, PHR may help characterize the association between tobacco-related metabolic disturbance and reproductive decline. This potential relationship, however, remains insufficiently studied.^[11–14]^

Leveraging large-scale data from the National Health and Nutrition Examination Survey (NHANES), this study aimed to systematically evaluate the independent associations of serum cotinine and PHR with women’s menopausal age and reproductive lifespan, further examine the statistical role of PHR in the association between cotinine and reproductive aging, and explore population-specific characteristics and quantitative patterns of these associations through subgroup analyses and threshold effect models.

## 2. Methods

### 2.1. Study population

**Figure 1.**
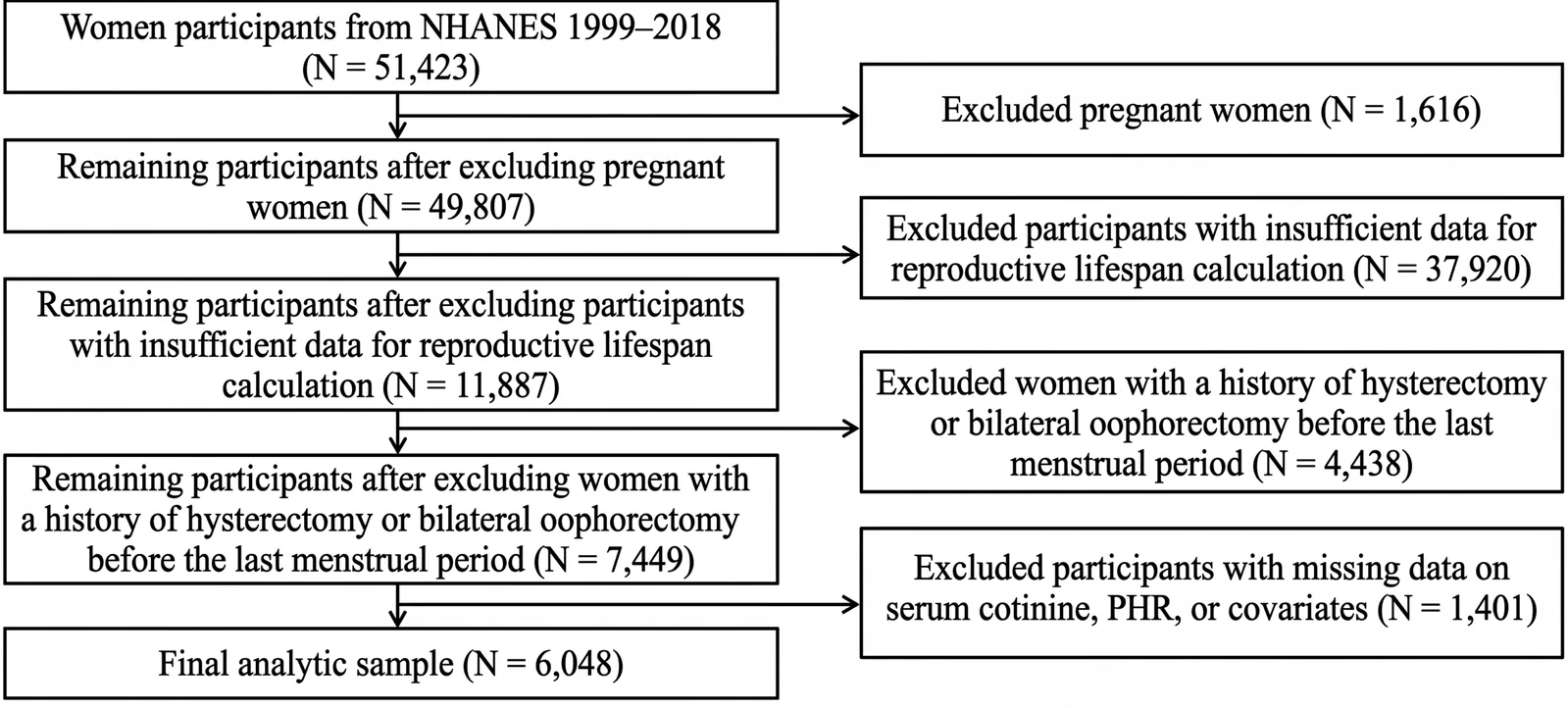
Process for including participants. NHANES = National Health and Nutrition Examination Survey, PHR = platelet-to-high-density lipoprotein cholesterol ratio.

This study analyzed data from the NHANES, a publicly accessible database spanning the 1999 to 2018 cycles (https://www.cdc.gov/nchs/nhanes/). NHANES employs a multistage stratified probability sampling design to generate nationally representative cohorts, incorporating comprehensive health assessments, including structured interviews, standardized physical examinations, and laboratory analyses. The survey protocol was approved by the National Center for Health Statistics Ethics Review Board, with all participants providing written informed consent. Given the use of deidentified, publicly available data, this study required no additional ethical clearance. From the initial NHANES cohort (1999–2018) comprising 51,423 women, 6048 eligible participants were included in the final analysis after sequential exclusions: 1616 pregnant individuals; 37,920 with insufficient reproductive lifespan data; 4438 who had undergone hysterectomy or bilateral oophorectomy; and 1402 with missing values for serum cotinine, PHR, or relevant covariates (Fig. 1). Participants with missing data on reproductive lifespan or other key covariates were excluded from the final analytic sample, and a complete-case analysis was conducted. No imputation procedures were applied for missing data.

### 2.2. Exposure measures

Serum cotinine concentrations were quantified by NHANES laboratories using isotope dilution-high-performance liquid chromatography-atmospheric pressure chemical ionization tandem mass spectrometry. Detailed protocols for detection limits, quality control, and standardization are available in official NHANES documentation (https://wwwn.cdc.gov/Nchs/Nhanes/2017-2018/COT_J.htm). The PHR was calculated as PLT count (×10^3^/μL) divided by HDL-C (mg/dL), where PLT was measured using a Beckman Coulter MAXM (Beckman Coulter, Inc.) automated hematology analyzer and HDL-C was determined via the high-density lipoprotein cholesterol 4 method.^[15]^

### 2.3. Outcome measures

Age at menarche and menopause were self-reported, and reproductive lifespan was calculated as the difference between these 2 ages.^[16]^

### 2.4. Covariates

Covariates were selected based on prior literature and included demographic factors (age: <60 years vs ≥60 years; race/ethnicity: Mexican American, Other Hispanic, non-Hispanic White, non-Hispanic Black, Other/multiracial; education level: <high school, high school/GED, >high school; marital status: married/living with partner, widowed/divorced/separated, never married; family poverty-to-income ratio [PIR]: ≤1.3, 1.3–3.5, >3.5), and lifestyle/clinical factors (body mass index [BMI]: <25, 25–29.9, ≥30 kg/m^2^; presence of diabetes; presence of hypertension; use of contraceptives; and use of hormone therapy).^[17]^

### 2.5. Statistical analysis

Statistical analyses were performed using R software (Version 4.3.3; R Foundation for Statistical Computing). NHANES data analysis adhered to the complex sampling design requirements. Because HDL-C was derived from the fasting subsample in the present analysis, the fasting subsample examination weight (WTSAF2YR) was applied. Because ten 2-year survey cycles (1999–2018) were combined, the original 2-year fasting subsample weights were divided by 10 to generate combined multi-cycle weights, in accordance with NHANES analytic guidance for pooled cycles. Continuous variables were reported as mean ± standard deviation, while categorical variables were reported as percentages. We divided serum cotinine concentration into 4 categories. Weighted linear regression models were employed to examine the associations of ln-transformed cotinine and PHR with reproductive aging parameters (menopausal age, reproductive lifespan, and menarche age), with adjustments for potential confounders. Subgroup analyses were conducted by stratifying participants according to demographic and clinical characteristics, with interaction terms incorporated into the regression models to evaluate the homogeneity of associations across subgroups. Restricted cubic spline (RCS) models were applied to explore potential nonlinear dose-response relationships between ln-transformed cotinine, ln-transformed PHR, and reproductive outcomes and to identify possible threshold effects. All statistical tests were two-tailed, with a *P*-value < .05 considered statistically significant.

## 3. Results

### 3.1. Baseline characteristics and intergroup comparisons

Our analysis of 6048 female participants from NHANES, stratified by serum cotinine quartiles (Q1: ≤0.011; Q2: 0.011–0.031; Q3: 0.031–0.207; Q4: >0.207 ng/mL), revealed significant demographic gradients (Table 1). Higher cotinine levels were associated with younger age distributions (Q4: 757 vs Q1: 562 participants aged <60 years; *P* < .001), increased representation of non-Hispanic Black individuals (395 vs 157), and inverse socioeconomic gradients in both educational attainment (higher education: 571 vs 1070) and PIR (≤1.3: 653 vs 452; all *P* < .001). Notably, the prevalence of diabetes (11.7%–13.3%) and hypertension (44.3%–49.9%) showed no significant differences across quartiles (*P* = .45 and 0.12, respectively).

**Table 1:** Baseline characteristics and intergroup comparisons.

Variables	Total	Serum cotinine, ng/mL				*P*
		Q1 (≤0.011)	Q2 (0.011–0.031)	Q3 (0.031–0.207)	Q4 (>0.207)	
N	6048	1916	1129	1493	1510	
Age, n (%)						<.001
<60 yr	2181 (45.9)	562 (38.9)	386 (42.8)	476 (42.5)	757 (61.0)	
≥60 yr	3867 (54.1)	1354 (61.1)	743 (57.2)	1017 (57.5)	753 (39.0)	
Race, n (%)						<.001
Mexican American	984 (4.9)	374 (5.7)	174 (4.8)	255 (4.8)	181 (3.9)	
Other Hispanic	572 (4.5)	228 (4.9)	127 (4.4)	114 (4.8)	103 (3.8)	
Non-Hispanic White	2986 (77.0)	1009 (81.5)	526 (75.3)	694 (73.5)	757 (75.1)	
Non-Hispanic Black	1031 (8.1)	157 (3.5)	188 (8.2)	291 (10.0)	395 (12.9)	
Other race – including multiracial	475 (5.5)	148 (4.4)	114 (7.3)	139 (7.0)	74 (4.4)	
Education, n (%)						<.001
<High school	1729 (16.6)	448 (10.1)	292 (13.8)	486 (20.5)	503 (24.1)	
High school grad/GED or equivalent	1466 (26.2)	398 (21.2)	255 (23.5)	377 (28.5)	436 (33.3)	
>High school	2853 (57.2)	1070 (68.7)	582 (62.7)	630 (51.1)	571 (42.6)	
PIR, n (%)						<.001
≤1.3	1897 (20.0)	452 (11.9)	300 (16.2)	492 (21.7)	653 (32.3)	
1.3–3.5	2319 (35.9)	726 (33.0)	431 (33.6)	590 (39.0)	572 (38.8)	
>3.5	1832 (44.2)	738 (55.1)	398 (50.3)	411 (39.2)	285 (28.9)	
Marital status, n (%)						<.001
Married/living with partner	3054 (57.9)	1069 (62.8)	614 (61.9)	723 (55.7)	648 (50.2)	
Widowed/divorced/separated	2523 (35.3)	720 (31.2)	449 (33.2)	674 (39.2)	680 (38.9)	
Never married	471 (6.8)	127 (6.0)	66 (4.9)	96 (5.0)	182 (10.9)	
BMI, n (%)						<.001
<25 kg/m^2^	1666 (30.6)	554 (31.4)	286 (29.4)	363 (26.4)	463 (34.1)	
25–29.9 kg/m^2^	1892 (30.5)	625 (31.7)	342 (30.1)	488 (32.7)	437 (27.2)	
≥30 kg/m^2^	2490 (38.9)	737 (36.9)	501 (40.6)	642 (40.9)	610 (38.7)	
Diabetes, n (%)	1044 (12.8)	316 (11.7)	203 (12.8)	268 (13.9)	257 (13.3)	.45
Hypertension, n (%)	3077 (45.7)	954 (44.3)	560 (44.7)	801 (49.9)	762 (44.8)	.120
Contraceptive use, n (%)	3564 (67.8)	1132 (69.1)	662 (67.5)	789 (60.5)	981 (72.7)	<.001
Hormone use, n (%)	1704 (33.9)	614 (38.0)	353 (36.9)	422 (35.2)	315 (24.7)	<.001
PHR, mean (SD)	179.25 (74.42)	168.42 (68.86)	176.86 (81.95)	179.57 (68.65)	195.81 (78.18)	<.001
Menarche age, yr, mean (SD)	12.80 (1.68)	12.74 (1.63)	12.79 (1.61)	12.97 (1.74)	12.74 (1.74)	.008
Menopausal age, yr, mean (SD)	47.31 (7.99)	48.63 (6.77)	48.07 (7.26)	47.75 (7.51)	44.52 (9.62)	<.001
Reproductive lifespan, yr, mean (SD)	34.51 (8.10)	35.89 (6.96)	35.28 (7.29)	34.78 (7.71)	31.78 (9.68)	<.001

BMI = body mass index, GED = General Educational Development, PIR = family poverty-to-income ratio, PHR = platelet-to-high-density lipoprotein cholesterol ratio, Q1–Q4 = quartile 1 to quartile 4, SD = standard deviation.

The highest cotinine quartile exhibited accelerated reproductive aging, manifesting as earlier menopause (44.52 ± 9.62 years vs 48.63 ± 6.77 years) and shorter reproductive lifespan (31.78 ± 9.68 years vs 35.89 ± 6.96 years; both *P* < .001), despite minimal variation in menarche age (12.74–12.97 years; *P* = .008 but clinically insignificant). This group showed paradoxical reproductive health patterns: highest contraceptive use (72.7% vs 69.1%) but lowest hormone therapy utilization (24.7% vs 38.0%). Most importantly, we identified a dose-dependent increase in PHR (195.81 ± 78.18 in Q4 vs 168.42 ± 68.86 in Q1; *P* < .001), suggesting that tobacco exposure may be linked to reproductive senescence through combined PLT activation and HDL-mediated metabolic dysfunction.

Table 2 presents the weighted linear regression analyses of ln-transformed cotinine and PHR levels in relation to reproductive aging parameters after adjustment for age, education, PIR, marital status, BMI, diabetes, hypertension, and the use of contraceptives or hormone therapy. Serum cotinine levels showed no significant association with menarche age (continuous β = −0.01, 95% confidence interval [CI]: −0.03 to 0.01, *P* = .163), but demonstrated significant inverse relationships with both menopausal age (β = −0.28, 95% CI: −0.35 to −0.20, *P* < .001) and reproductive lifespan (β = −0.26, 95% CI: −0.34 to −0.18, *P* < .001). Quartile analyses revealed that compared with the lowest quartile (Q1), women in the highest cotinine quartile (Q4) experienced earlier menopause (β = −2.39 years, 95% CI: −3.06 to −1.72) and shorter reproductive lifespan (β = −2.36 years, 95% CI: −3.06 to −1.65) (both *P* < .001). Similarly, PHR levels showed significant negative associations with menopausal age (β = −1.86, 95% CI: −2.58 to −1.13) and reproductive lifespan (β = −1.91, 95% CI: −2.66 to −1.16) (both *P* < .001), with the highest PHR quartile (Q4) associated with menopause 2.28 years earlier (95% CI: −3.11 to −1.45) and a reproductive lifespan 2.32 years shorter (95% CI: −3.18 to −1.45) compared with Q1 (both *P* < .001). Significant dose-response relationships were observed for both biomarkers with menopausal age and reproductive lifespan (*P* for trend < .001 for all associations).

**Table 2:** Associations of serum cotinine and PHR with reproductive aging parameters in weighted linear regression models.

Variables	Menarche age		Menopausal age		Reproductive lifespan	
	β (95% CI)	*P*	β (95% CI)	*P*	β (95% CI)	*P*
Ln-cotinine						
Continuous	−0.01 (−0.03 to 0.01)	.163	−0.28 (−0.35 to −0.2)	<.001	−0.26 (−0.34 to −0.18)	<.001
Q1	Reference		Reference		Reference	
Q2	0.04 (−0.11 to 0.19)	.622	−0.34 (−1.07 to 0.39)	.353	−0.37 (−1.1 to 0.36)	.317
Q3	0.19 (0.03–0.35)	.024	−0.47 (−1.09 to 0.15)	.136	−0.64 (−1.29 to 0.01)	.047
Q4	−0.04 (−0.23 to 0.15)	.7	−2.39 (−3.06 to −1.72)	<.001	−2.36 (−3.06 to −1.65)	<.001
*P* for trend	.324		<.001		<.001	
Ln-PHR						
Continuous	0.05 (−0.08 to 0.19)	.428	−1.86 (−2.58 to −1.13)	<.001	−1.91 (−2.66 to −1.16)	<.001
Q1	Reference		Reference		Reference	
Q2	0.08 (−0.1 to 0.26)	.389	−0.52 (−1.22 to 0.19)	.147	−0.6 (−1.31 to 0.11)	.098
Q3	0.19 (0.03–0.36)	.02	−0.55 (−1.3 to 0.19)	.144	−0.75 (−1.53 to 0.03)	.061
Q4	0.04 (−0.12 to 0.2)	.62	−2.28 (−3.11 to −1.45)	<.001	−2.32 (−3.18 to −1.45)	<.001
*P* for trend	.333		<.001		<.001	

Models were adjusted for age, education, PIR, marital status, BMI, diabetes, hypertension, and use of contraceptives or hormone therapy. “*P* for trend” values assess linear trends across ordered quartiles (Q1–Q4) of serum cotinine or PHR. β represents the regression coefficient.

BMI = body mass index, CI = confidence interval, PIR = family poverty-to-income ratio, PHR = platelet-to-high-density lipoprotein cholesterol ratio.

**Figure 2.**
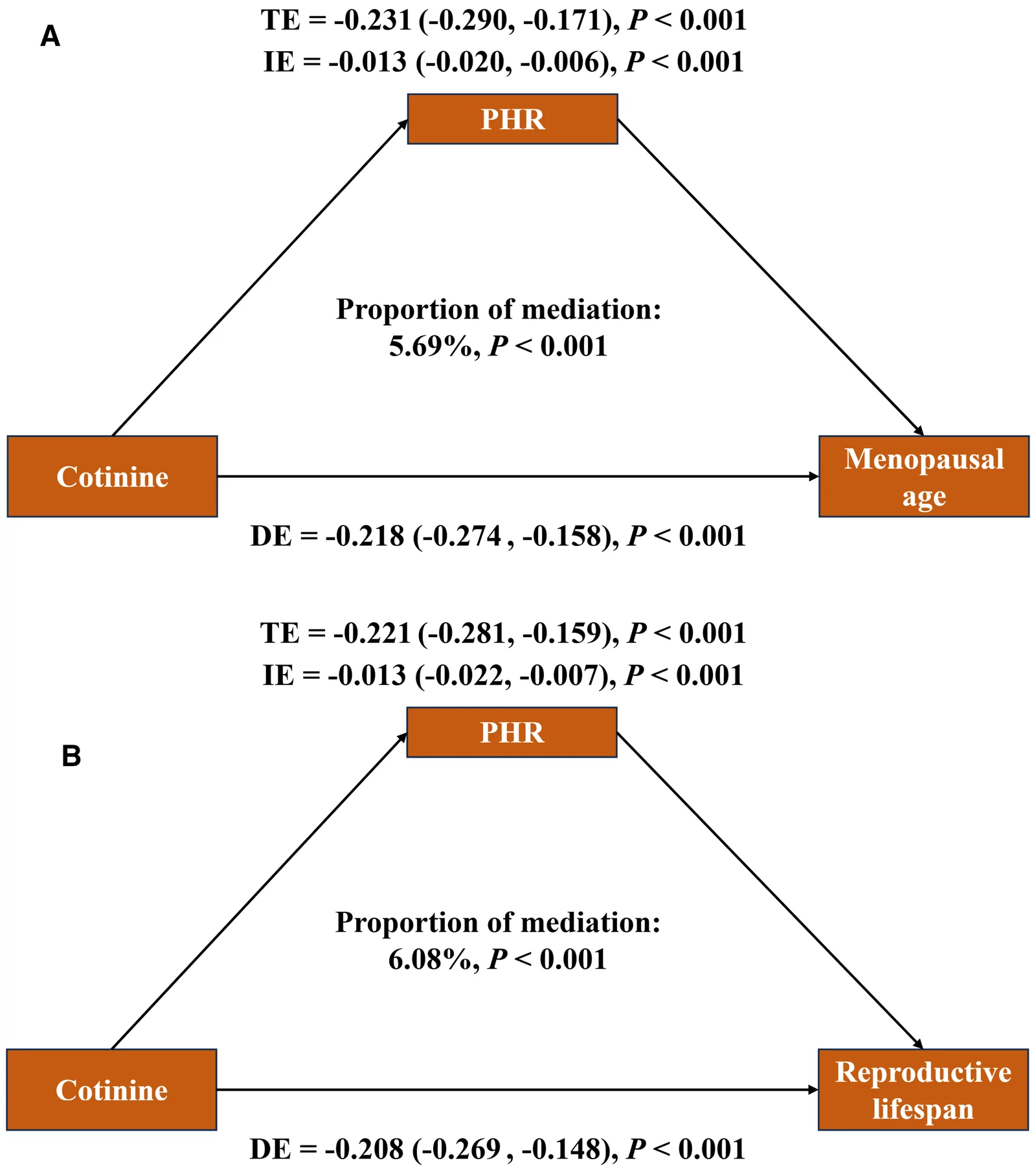
Exploratory statistical mediation analysis of PHR. Panels (A) and (B) correspond to the statistically mediated associations involving PHR in the relationship between cotinine and menopausal age, and between cotinine and reproductive lifespan, respectively. TE represents total effect, IE represents indirect effect, and DE represents direct effect. PHR = platelet-to-high-density lipoprotein cholesterol ratio.

Figure 2 presents the exploratory statistical mediation analyses involving PHR. In Figure 2A, serum cotinine showed a significant direct effect on menopausal age (β = −0.218, 95% CI: −0.274 to −0.158, *P* < .001), with a total effect of −0.231 (95% CI: −0.290 to −0.171, *P* < .001). The indirect effect mediated through PHR was −0.013 (95% CI: −0.020 to −0.006, *P* < .001), accounting for 5.69% of the observed association. Similarly, Figure 2B presents the corresponding mediation analysis for reproductive lifespan. The direct effect of cotinine on reproductive lifespan was −0.208 (95% CI: −0.269 to −0.148, *P* < .001), while the total effect was −0.221 (95% CI: −0.281 to −0.159, *P* < .001). The indirect effect through PHR was −0.013 (95% CI: −0.022 to −0.007, *P* < .001), accounting for 6.08% of the observed association. These findings indicate that PHR accounted for a significant but modest proportion of the associations between serum cotinine and reproductive aging outcomes.

### 3.2. Subgroup analysis

As shown in Table 3, age demonstrated significant interactions with both menopausal age (*P* for interaction < .001) and reproductive lifespan (*P* for interaction < .001). The <60 years group exhibited stronger inverse associations for menopausal age (β = −0.41, 95% CI: −0.52 to −0.29, *P* < .001) and reproductive lifespan (β = −0.37, 95% CI: −0.49 to −0.26, *P* < .001) compared with the ≥60 years group (menopausal age: β = −0.09, 95% CI: −0.18 to −0.00, *P* = .042; reproductive lifespan: β = −0.10, 95% CI: −0.19 to −0.02, *P* = .022). Race showed significant interactions with menopausal age (*P* = .003) and reproductive lifespan (*P* = .003), with only non-Hispanic White participants presenting significant inverse associations (menopausal age: β = −0.32, 95% CI: −0.42 to −0.23, *P* < .001; reproductive lifespan: β = −0.31, 95% CI: −0.41 to −0.22, *P* < .001), while other racial groups had no significant associations. Education showed no significant interactions with menopausal age (*P* = .826) or reproductive lifespan (*P* = .680), as inverse associations were observed across all education levels (e.g., >high school group: menopausal age β = −0.30, *P* < .001; reproductive lifespan β = −0.29, *P* < .001). For PIR, the 1.3 to 3.5 group displayed the strongest inverse associations (menopausal age: β = −0.36, 95% CI: −0.49 to −0.22, *P* < .001; reproductive lifespan: β = −0.34, 95% CI: −0.48 to −0.20, *P* < .001), with no significant interaction (*P* = .108 for menopausal age; *P* = .166 for reproductive lifespan). Marital status showed near-significant interactions with menopausal age (*P* = .064) and reproductive lifespan (*P* = .065), with the never married group exhibiting the strongest inverse associations (menopausal age: β = −0.40, 95% CI: −0.73 to −0.08, *P* = .017; reproductive lifespan: β = −0.37, 95% CI: −0.72 to −0.03, *P* = .034).

**Table 3:** Subgroup analyses of the associations of ln-serum cotinine with menopausal age and reproductive lifespan.

Characteristic	Menopausal age			Reproductive lifespan		
	β (95% CI)	*P*	*P* for interaction	β (95% CI)	*P*	*P* for interaction
Age			<.001			<.001
<60 yr	−0.41 (−0.52 to −0.29)	<.001		−0.37 (−0.49 to −0.26)	<.001	
≥60 yr	−0.09 (−0.18 to −0.00)	.042		−0.10 (−0.19 to −0.02)	.022	
Race			.003			.003
Mexican American	−0.09 (−0.31 to 0.13)	.422		−0.07 (−0.30 to 0.15)	.539	
Other Hispanic	−0.18 (−0.47 to 0.11)	.219		−0.14 (−0.45 to 0.17)	.366	
Non-Hispanic White	−0.32 (−0.42 to −0.23)	<.001		−0.31 (−0.41 to −0.22)	<.001	
Non-Hispanic Black	−0.07 (−0.21 to 0.07)	.328		−0.06 (−0.21 to 0.09)	.45	
Other race – including multiracial	0.04 (−0.21 to 0.30)	.747		0.10 (−0.17 to 0.38)	.476	
Education			.826			.68
<High school	−0.26 (−0.41 to −0.11)	<.001		−0.22 (−0.37 to −0.06)	.006	
High school grad/GED or equivalent	−0.24 (−0.42 to −0.06)	.009		−0.24 (−0.42 to −0.06)	.009	
>High school	−0.30 (−0.42 to −0.18)	<.001		−0.29 (−0.41 to −0.17)	<.001	
PIR			.108			.166
≤1.3	−0.25 (−0.38 to −0.12)	<.001		−0.22 (−0.36 to −0.08)	.002	
1.3–3.5	−0.36 (−0.49 to −0.22)	<.001		−0.34 (−0.48 to −0.20)	<.001	
>3.5	−0.16 (−0.31 to −0.02)	.025		−0.16 (−0.30 to −0.02)	.024	
Marital status			.064			.065
Married/living with partner	−0.29 (−0.40 to −0.18)	<.001		−0.26 (−0.37 to −0.15)	<.001	
Widowed/divorced/separated	−0.24 (−0.33 to −0.15)	<.001		−0.25 (−0.35 to −0.15)	<.001	
Never married	−0.40 (−0.73 to −0.08)	.017		−0.37 (−0.72 to −0.03)	.034	
BMI			.954			.993
<25 kg/m^2^	−0.28 (−0.41 to −0.15)	<.001		−0.26 (−0.40 to −0.13)	<.001	
25–29.9 kg/m^2^	−0.29 (−0.43 to −0.16)	<.001		−0.25 (−0.39 to −0.12)	<.001	
≥30 kg/m^2^	−0.26 (−0.40 to −0.12)	<.001		−0.26 (−0.40 to −0.13)	<.001	
Diabetes			<.001			<.001
Yes	−0.15 (−0.25 to −0.05)	.004		−0.14 (−0.24 to −0.04)	.009	
No	−0.38 (−0.49 to −0.27)	<.001		−0.36 (−0.47 to −0.26)	<.001	
Hypertension			.044			.06
Yes	−0.20 (−0.40 to −0.01)	.045		−0.20 (−0.39 to −0.01)	.039	
No	−0.29 (−0.37 to −0.21)	<.001		−0.27 (−0.36 to −0.19)	<.001	
Contraceptive use			.487			.585
Yes	−0.26 (−0.35 to −0.16)	<.001		−0.25 (−0.34 to −0.15)	<.001	
No	−0.32 (−0.46 to −0.18)	<.001		−0.30 (−0.44 to −0.15)	<.001	
Hormone use			.278			.375
Yes	−0.25 (−0.37 to −0.12)	<.001		−0.24 (−0.38 to −0.11)	<.001	
No	−0.29 (−0.39 to −0.19)	<.001		−0.27 (−0.37 to −0.17)	<.001	

Models were adjusted for the same covariates as in Table 2, except for the stratification variable in each subgroup analysis.

BMI = body mass index, CI = confidence interval, GED = General Educational Development, PIR = family poverty-to-income ratio.

BMI showed no significant interactions with menopausal age (*P* = .954) or reproductive lifespan (*P* = .993), as all BMI subgroups displayed significant inverse associations (e.g., <25 kg/m^2^ group: menopausal age β = −0.28, *P* < .001; reproductive lifespan β = −0.26, *P* < .001). Diabetes had significant interactions with menopausal age (*P* < .001) and reproductive lifespan (*P* < .001), with stronger inverse associations in the nondiabetic group (menopausal age: β = −0.38, 95% CI: −0.49 to −0.27, *P* < .001; reproductive lifespan: β = −0.36, 95% CI: −0.47 to −0.26, *P* < .001) compared with the diabetic group (menopausal age: β = −0.15, *P* = .004; reproductive lifespan: β = −0.14, *P* = .009). Hypertension interacted significantly with menopausal age (*P* = .044), with a stronger inverse association in the non-hypertensive group (β = −0.29, 95% CI: −0.37 to −0.21, *P* < .001) versus the hypertensive group (β = −0.20, 95% CI: −0.40 to −0.01, *P* = .045); the interaction with reproductive lifespan was near-significant (*P* = .060). Contraceptive use (*P* = .487 for menopausal age; *P* = .585 for reproductive lifespan) and hormone use (*P* = .278 for menopausal age; *P* = .375 for reproductive lifespan) showed no significant interactions, with consistent inverse associations across all subgroups.

### 3.3. RCS analyses

**Figure 3.**
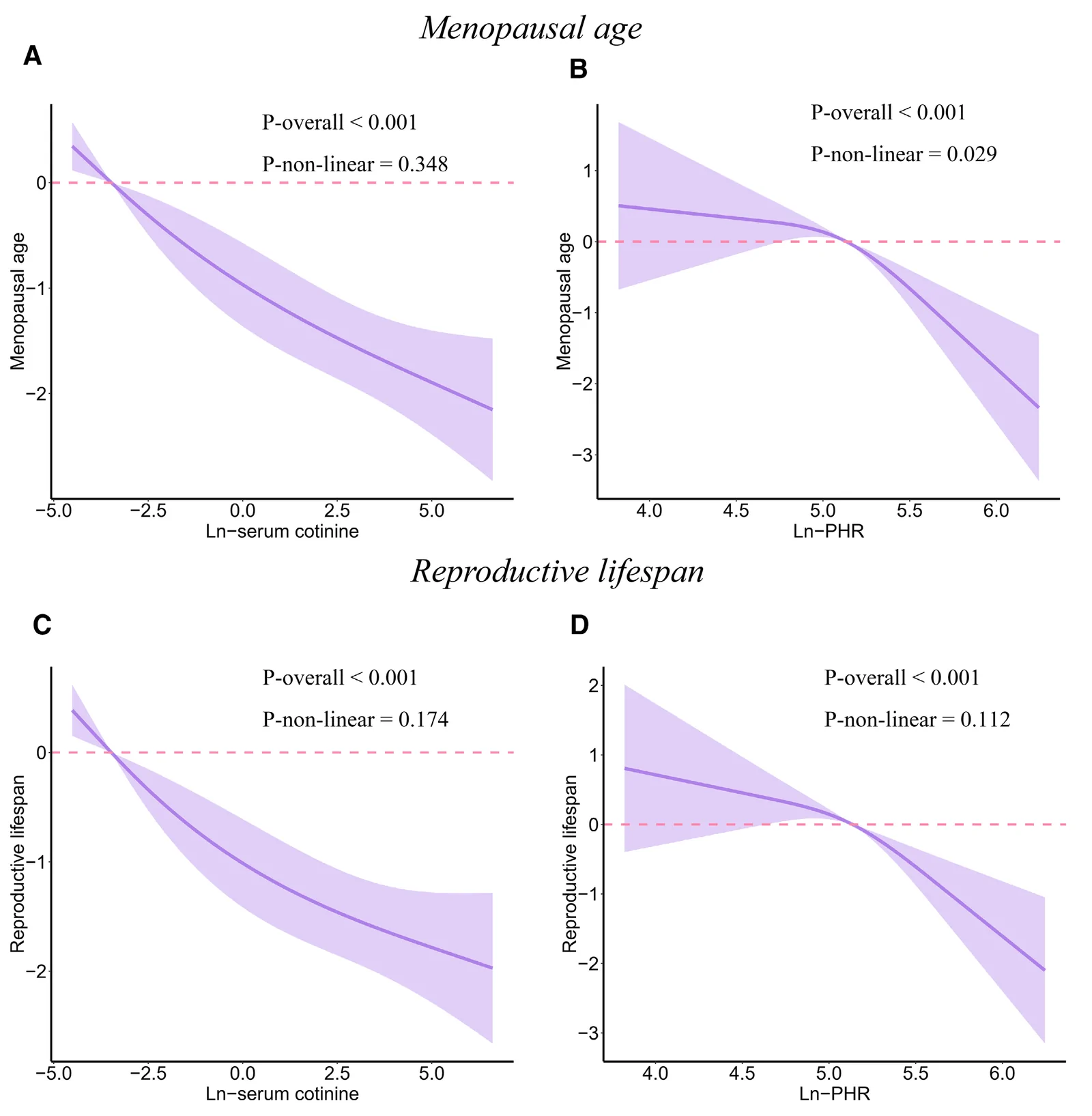
Multivariable-adjusted RCS analyses. Both cotinine and PHR were ln-transformed. Panels (A, B) and (C, D) correspond to the dose-response relationships of cotinine and PHR with the outcome variables (menopausal age and reproductive lifespan), respectively. The purple line represents the fitted regression coefficients (β), and the light purple shading denotes the 95% confidence intervals. *P*-overall indicates the overall association between the exposure factor and the outcome; *P*-nonlinear denotes the nonlinear relationship between the exposure factor and the outcome. PHR = platelet-to-high-density lipoprotein cholesterol ratio, RCS = restricted cubic spline.

RCS analysis with multivariable adjustment (Fig. 3) further characterized the dose-response relationships between ln-cotinine, ln-PHR, and reproductive parameters. The exposure-response curves in Figure 3A to D exhibited a consistent trend, where the fitted β coefficient shifted from positive to negative with increasing exposure levels, suggesting possible threshold-like patterns rather than confirming uniform threshold effects across all outcomes. Statistical analyses revealed that the overall associations between ln-cotinine/ln-PHR and the outcomes (menopausal age, reproductive lifespan) were statistically significant (all *P* < .001). In the nonlinearity tests, only the association between PHR and menopausal age reached significance (*P* = .029), while the *P*-values for the remaining nonlinearity tests were all >.100.

Stratified threshold effect analysis (Table 4) showed that the inflection point of ln-cotinine was −3.47 in both the analyses of menopausal age and reproductive lifespan. Below this threshold, cotinine exhibited a nonsignificant inverse trend (menopausal age: β = −0.59, *P* = .103; reproductive lifespan: β = −0.55, *P* = .140). In contrast, above this threshold, cotinine showed significant adverse associations (menopausal age: β = −0.19, *P* < .001; reproductive lifespan: β = −0.17, *P* < .001). For PHR, the inflection points varied by outcome (menopausal age: 4.59; reproductive lifespan: 5.13). Below the inflection points, PHR had no significant effect (menopausal age: β = 2.58, *P* = .147; reproductive lifespan: β = −0.70, *P* = .197), whereas above the inflection points, it showed significant adverse associations (menopausal age: β = −1.76, *P* < .001; reproductive lifespan: β = −2.42, *P* < .001). A statistically significant threshold effect was observed only for PHR in relation to menopausal age (likelihood ratio test, *P* = .023), whereas the threshold effects for other associations were not statistically significant (*P* > .100).

**Table 4:** Stratified threshold effect analyses.

Outcome	Parameter	Ln-cotinine		Ln-PHR	
		β (95% CI)	*P*	β (95% CI)	*P*
Menopausal age	Inflection point	−3.47		4.59	
	Below inflection point	−0.59 (−1.30, 0.12)	.103	2.58 (−0.89, 6.04)	.147
	Above inflection point	−0.19 (−0.27, −0.10)	<.001	−1.76 (−2.38, −1.15)	<.001
	*P* for likelihood test		.631		.023
Reproductive lifespan	Inflection point	−3.47		5.13	
	Below inflection point	−0.55 (−1.28, 0.18)	.140	−0.70 (−1.77, 0.36)	.197
	Above inflection point	−0.17 (−0.25, −0.08)	<.001	−2.42 (−3.67, −1.17)	<.001
	*P* for likelihood test		.643		.102

The inflection point was defined as the value of the exposure variable at which the fitted β coefficient crossed 0 in the restricted cubic spline analysis.

CI = confidence interval, PHR = platelet-to-high-density lipoprotein cholesterol ratio.

## 4. Discussion

In this cross-sectional analysis of NHANES data, higher serum cotinine and higher PHR were associated with earlier menopause and a shorter reproductive lifespan. PHR also accounted for a modest statistical proportion of the association between cotinine and reproductive aging indicators. These findings support the possibility that tobacco exposure and metabolic-inflammatory status are jointly related to reproductive aging; however, the observational and cross-sectional design does not allow causal inference, and our findings should therefore be interpreted with appropriate caution.

Serum cotinine, a validated biomarker of tobacco exposure, has been associated with adverse reproductive aging outcomes and may plausibly operate through both direct toxic effects and broader metabolic or inflammatory pathways. Prior experimental and epidemiological evidence suggests that tobacco-derived toxins, including nicotine and polycyclic aromatic hydrocarbons, may reach ovarian tissue through the circulation and contribute to granulosa cell apoptosis and accelerated follicular depletion.^[18]^ Such processes could reduce both follicle quantity and quality, thereby contributing to earlier menopause and a shorter reproductive lifespan.^[19]^ Tobacco exposure has also been linked to the disruption of hypothalamic-pituitary-ovarian axis homeostasis.^[20–22]^

The statistically mediated proportion attributed to PHR was modest, and this finding should be interpreted as indicating a possible link between tobacco exposure and metabolic dysregulation rather than demonstrating a confirmed biological pathway.^[23]^ Tobacco exposure may influence PLT activity and lipid metabolism in ways that increase PHR, but the present study cannot determine the directionality or mechanistic sequence of these relationships.^[24]^ The observed increase in PHR across cotinine quartiles is therefore best understood as supportive of biological plausibility while still requiring validation in prospective and mechanistic studies.

In refining this interpretation, elevated PHR may reflect a state of PLT-related inflammatory activation and HDL-C dysfunction that is relevant to reproductive aging, rather than serving as direct proof of a mechanistic pathway. PLTs, classically characterized by their hemostatic functions but increasingly recognized as immunomodulatory sentinels, can amplify inflammatory cascades via direct immune cell engagement or paracrine signaling.^[25]^ Within the ovarian stroma, activated PLTs secrete a variety of growth factors (e.g., transforming growth factor beta and platelet-derived growth factor) and cytokines that may exacerbate local oxidative stress and tissue remodeling, whereas reduced HDL-C function may weaken anti-inflammatory and antioxidative buffering.^[26,27]^ Taken together, these observations provide biological plausibility for the statistical associations observed in our study, but they should be interpreted as hypothesis-supporting rather than causal evidence.^[28]^

Notably, the observed pattern of cotinine, PHR, and reproductive aging also showed heterogeneity across population subgroups, further highlighting the complexity of interactions between tobacco exposure and metabolic dysregulation. Among clinical subgroups, metabolic status appeared to modulate association strengths. In nondiabetic individuals, cotinine’s negative associations with menopausal age (β = −0.38) and reproductive lifespan (β = −0.36) were stronger than those observed in participants with diabetes; a similar pattern was seen for hypertension status. These subgroup differences may reflect variation in background metabolic risk, treatment patterns, or competing sources of inflammation, but such interpretations remain speculative and require confirmation in future studies.

These findings may still have potential clinical and public health relevance, but they should primarily be interpreted as observational associations rather than direct evidence for intervention targets or treatment effects.^[29,30]^ The observed thresholds may help generate hypotheses for future risk stratification, and lifestyle factors such as smoking cessation, healthy diet, regular exercise, and weight control remain reasonable targets for general health promotion.^[31,32]^ Whether pharmacological or lifestyle interventions that influence HDL metabolism or PLT activity can modify reproductive aging trajectories remains unknown and warrants prospective investigation.^[33,34]^

Translating these findings to practice, PHR may be considered a convenient candidate marker for further study because it can be derived from routine complete blood counts and lipid profiles. However, its utility in clinical screening or risk prediction for reproductive aging has not yet been established.^[35,36]^ Future studies should evaluate whether incorporating PHR into reproductive health assessment frameworks improves prediction beyond conventional indicators and whether the observed subgroup heterogeneity can be replicated in other populations.

This study has several notable strengths. First, the analysis was based on a sample of 6048 participants from NHANES, providing broad national coverage and supporting the generalizability of the findings within the US population. Second, we adjusted for multiple demographic and clinical covariates, which helped reduce confounding to some extent. Third, the combined use of quartile-based analyses and RCS models allowed us to evaluate both linear associations and possible threshold effects. Nevertheless, these strengths should be interpreted alongside the study limitations, particularly the cross-sectional design and the modest magnitude of the mediation proportion.

However, several limitations should be acknowledged. First, the cross-sectional design precludes establishing temporal relationships between variables and limits causal inference. Second, cotinine and PHR were measured at the NHANES examination, whereas age at menopause was retrospectively reported and may have occurred years earlier; therefore, the temporal ordering required for mediation analysis cannot be firmly established. Third, although we adjusted for multiple demographic and clinical factors, some potentially important confounders, such as alcohol consumption, physical activity, dietary patterns, and reproductive history variables, including parity, were not fully accounted for in the present study. Therefore, residual confounding cannot be excluded. In addition, because a large number of participants were excluded due to missing reproductive lifespan information or other key variables, selection bias cannot be ruled out. Finally, the use of self-reported reproductive history may have introduced recall bias.

## 5. Conclusion

Our study suggests that PHR may be a useful biomarker associated with female reproductive aging. Higher serum cotinine and PHR were associated with earlier menopause and shorter reproductive lifespan, and PHR showed a modest statistically mediated proportion in these associations. The observed thresholds and subgroup heterogeneity may be useful for hypothesis generation and future risk stratification, but prospective or longitudinal studies are needed to validate these findings and to clarify the potential role of PHR in reproductive aging.

## Acknowledgments

We would like to thank all participants in this study.

## Author contributions

**Writing – original draft:** Jie Liao, De Qiong, Dawa Zhuoma, Weiwei Ning, Da Ga, Silang Quncuo, Jing Jing, Lingxiu Li, Yalan Li, Xin Li, Bin Lv, Hengxi Chen.

**Writing – review & editing:** Jie Liao, De Qiong, Dawa Zhuoma, Weiwei Ning, Da Ga, Silang Quncuo, Jing Jing, Lingxiu Li, Yalan Li, Xin Li, Bin Lv, Hengxi Chen.
